# The experience of patients with cancer on narrative practice: A systematic review and meta‐synthesis

**DOI:** 10.1111/hex.13003

**Published:** 2020-01-16

**Authors:** Yan Yang, Jiehui Xu, Yihui Hu, Jiale Hu, Anli Jiang

**Affiliations:** ^1^ Department of Humanistic Nursing School of Nursing Second Military Medical University Shanghai China; ^2^ Department of Breast Surgery School of Medicine Renji Hospital Shanghai Jiaotong University Shanghai China; ^3^ Department of Nursing School of Medicine Renji Hospital Shanghai Jiaotong University Shanghai China; ^4^ College of Health Professions Virginia Commonwealth University Richmond VA USA

**Keywords:** experience, meta‐synthesis, narrative practice, patients with cancer, systematic review

## Abstract

**Background:**

In recent years, narrative practice has been applied in clinical settings to address the relational and psychological concerns that occur in tandem with physical illness. It is an emerging strategy to treat patients as individuals with their own stories, rather than purely based on symptoms.

**Objective:**

To synthesize the experience of patients with cancer using narrative practice.

**Methods:**

Following a systematic search strategy, a literature search was conducted to identify qualitative studies on the experience of patients with cancer using narrative practice. Nine databases were searched up to April 2018, which included six English databases and three Chinese databases. A meta‐synthesis was conducted to synthesize the findings of the included studies.

**Main Results:**

Seven studies out of 2894 studies were included in this review. Patients with cancer had different preferences on narrative practices. In terms of the impacts of narrative practice on patients with cancer, six themes were identified, which included ‘(a) reducing the gap between patients and clinicians; (b) healing effect; (c) social connection; (d) facilitating self‐reflection, self‐recognition and self‐realization; (e) risk of negative impacts; and (f) Patients' preference on different approaches of narrative practice’.

**Conclusions:**

Patients with cancer experienced positive effects regarding narrative practice. Although some patients may experience negative effects, narrative practice is a humanized way to provide care for patients with cancer in the clinical settings.

## 1 INTRODUCTION

The number of patients with cancer has increased tremendously in recent years.^1^ The Global Cancer Statistics 2018 estimated 18.1 million new cancer cases and 9.6 million cancer deaths in 2018.^2^ According to the National Health and Family Planning Commission of China, there were an estimated 42.92 million new cancer cases in China in 2015.^3^ In the United States of America, 17.62 million new cancer cases and 0.61 cancer deaths are projected in 2019.^4^

Patients with cancer experience many problems in the course of diagnosis, treatment and survival. The problems include delays and lack of coordination of care, information gap and not being actively engaged in management, inadequate attention to their emotional and social problems, and difficulty in accessing services due to financial problems.^5^ Studies show cancer patients experience difficult emotions and have more psychological concerns, anxiety, depression, unmet requirements and more concerns about finance, jobs and self‐image than patients without cancer.^5^, ^6^ The amount of concerns experienced are related to their quality of life.^6^ Therefore, their thoughts and concerns are not only important to them but also to health‐care providers in clinical practice.

Health‐care providers have tried numerous ways to help patients speak out and release their thoughts. It is well documented that nursing scholars, like Nightingale and Watson, provided caring through the use of media, such as art, music and writing.^7^, ^8^ These are the prototypes of narrative practice. This shows that narrative practice has a long history in medical field. ‘A narrative or story is a report of connected events, real or imaginary, presented in a sequence of written or spoken words, or still or moving images, or both’.^9^ Narrative medicine is the most prominent recent development in the medical humanities.^10^ Rita Charon suggests that narrative medicine is a commitment to understanding patients' lives, caring for the caregivers and giving voice to the suffering.^11^ Narrative medicine is a medical approach that utilizes people's narratives in clinical practice, research and education as a way to promote healing.^11^, ^12^ It aims to address the relational and psychological issues that occur in tandem with physical illness, and attempt to treat patients as humans with individual stories, rather than purely based on symptoms.^13^ Its central claim is that attention to patients' narratives is essential for patient care.^13^ There are many approaches and different types of narrative practice. They include telling illness stories, writing personal experiences through letters or blogs,^10^ taking part in sharing conference of patients,^14^ drawing,^11^ reading or listening or watching others' stories.^12^

The significance of the patients' narrative of illness provides insight into their experiences.^15^ Fraas summarized the importance of narratives throughout the course of treatment. During the process of diagnosis, narratives encourage empathy and promote mutual understanding between health‐care providers and patients.^16^ During the process of treatment, it provides useful information in decision making and disease management. Moreover, narratives encourage reflection on the behaviour and thoughts.^16^ Thus, mutual understanding could be fostered, not only from the medical side to their patients but also from patients to their caregivers.

Narrative practice has shown significant influence on patients in quantitative studies. For instance, narratives in diabetes patients facilitate health behaviour changes, self‐efficacy and self‐care activities.^14^ Results from a survey suggested that narrative blogging may decrease a sense of isolation through developing online connections with others and increases a sense of helping others in similar situations.^17^ Interestingly, a systematic review concluded that there were no significant differences regarding psychological health indexes by utilizing writing intervention at any of the follow‐up time‐points.^18^ Although quantitative evidence has shown the benefits of narrative practice to patients, what patients experience in the process of storytelling or writing or how they think of narrative practice is still not synthesized. Furthermore, the target population in these quantitative studies normally included patients with diabetes, with pain, in intensive care units, and so forth. Patients with cancer have yet to draw the attention of the researchers in the domain of narrative medicine.

Thus, the purpose of this review was to synthesize the experience of patients with cancer using narrative practice. There is a lack of published meta‐syntheses of findings concerning the experience of narrative practice for cancer patients, and this systematic review and meta‐syntheses could serve as a reference for further studies in this field.

## 2 METHODS

### 2.1 Design

This systematic review synthesized the experience of cancer patients regarding narrative practice reported in qualitative studies, as well as in mixed‐methods research papers.

### 2.2 Protocol and registration

A draft research protocol was discussed and revised in the author group. The senior author (J.H) and a research librarian (MD) reviewed the protocol, and revisions made as necessary. The research protocol was registered in PROSPERO (2017: CRD42017078900).

### 2.3 Inclusion criteria

Studies were included if they (a) included adult patients with cancer, (b) used a qualitative design (interviews, focus groups or written text description analysis) and (c) focused on patients' experience and perspectives on narrative practice. Studies were excluded if they (a) did not focus on narrative practice, (b) used a quantitative design, (c) not published in English and/or Chinese. Studies using a mixed‐methods design were included only if data obtained via qualitative techniques could be (or were) disaggregated, and if qualitative data fulfilled the above‐mentioned inclusion criteria.

### 2.4 Search method

A three‐step search strategy was applied in this review. An initial scoping search of EMBASE and MEDLINE was undertaken to analyse the key words contained in the title and abstract and subject headings associated with preliminary articles of interest which could be used to help identify additional articles of relevance. A second search using all identified terms was then undertaken across all included databases and other resources. The search strategy was developed by two authors (J.X and Y.H) with the librarian (MD) experienced in systematic review searching and peer‐reviewed by a second librarian (TT). Thirdly, the list of references of all identified articles and studies was searched and reviewed for additional eligible studies.

Searches of the following databases were used to identify relevant literature: (a) English database—(1) MEDLINE including In‐Process & Other Non‐Indexed Citations (1946 to present), (2) EMBASE (1947 to present) and (3) PsycINFO (1806 to present); (b) Chinese database—(1) China Academic Journal Network Publishing Database, (2) China National Knowledge Infrastructure (CNKI) and (3) China Science and Periodical Database. Other literature resource included was as follows: ProQuest Dissertations & Theses Global; Web of Science; Cochrane Library. Main search strategies in different databases are listed in the Appendix S1.

### 2.5 Screening

After duplicates were removed from the collection of citations identified from the electronic search, a two‐step screening was conducted by two reviewers (J.X and Y.H). In the first step, the two reviewers independently screened the titles and abstracts of all studies to determine their potential eligibility for the review. All potentially relevant records and those records that did not contain enough information to determine eligibility (eg no available abstract) were retained. In the second step, the same two reviewers independently read the full text of all potentially relevant articles to determine eligibility for inclusion in the review. The reviewers met at the beginning, mid‐point and final stages of each step to discuss challenges and uncertainties related to study selection. Any conflicts in the identification of relevant studies during either the first or second step were resolved through a consensus process or by consulting a third senior reviewer, as required. Reasons for exclusion were documented for all papers excluded from the review at this second step.

### 2.6 Data extraction

Data were extracted from studies included in this systematic review using extraction tables. Two authors (J.X and Y.H) independently extracted data from eligible articles. The data included participants and setting, aims or purpose of the study, research design, sampling, data collection and analysis methods, findings and themes relevant to the review. Discrepancies in data extraction were resolved through consensus. The characteristics of the included studies were integrated into extraction tables, and the data were transferred into NVivo 12.0^19^ for further synthesis.

### 2.7 Risk of bias (quality) assessment

The Joanna Briggs Institute Critical Appraisal tool (Checklist for Qualitative Research) was utilized for the judgement of the studies included.^20^ Two reviewers (J.X and Y.H) independently assessed the study quality and the risk of bias. If the agreement was not achieved, the two reviewers consulted the third reviewer (J.H) to make a final decision.

### 2.8 Data synthesis

Thematic synthesis was conducted to analyse the qualitative data in the included studies. It involved the systematic coding of data, generating descriptive and analytical themes.^21^ Such a synthesis can produce new insights and understanding as studies with different important aspects, such as population and settings, can be combined to identify concepts that are present across a range of contexts and settings. The synthesis included three stages: the free line‐by‐line coding of the findings of primary studies; the organization of these ‘free codes’ into related areas to construct ‘descriptive’ themes; and the development of ‘analytical’ themes.^22^

The preliminary findings were discussed with another author (J.H) with experience in qualitative research. The descriptive and analytical themes and their distinctions from each other were considered, afterwards some thematic themes were grouped together and reworded.^22^

## 3 RESULTS

### 3.1 Study selection

**Figure 1:**
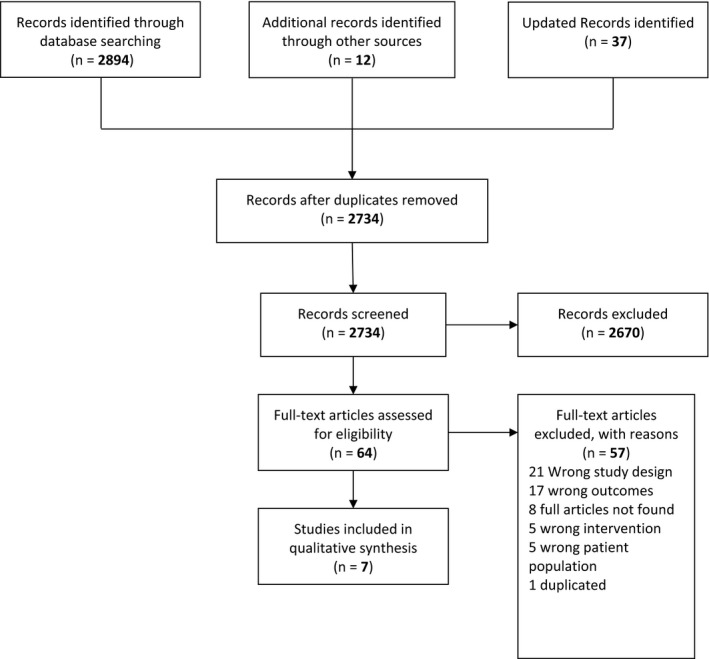
Flow diagram of study selection

A total of 2894 studies were identified and imported into EndNote X7 software.^23^ From this sample, 160 duplications were removed. The remaining 2734 studies were assessed for relevance of title and abstract. From this pool, 64 full texts were retrieved and reviewed independently by two reviewers (J.X and Y.H) according to the inclusion criteria. Finally, a total of seven articles were included for data synthesis and quality appraisal (Figure 1).

### 3.2 Characteristics of the included data with the tables

Of the seven studies included for final analysis, six were qualitative studies and one mixed‐method study (Table 1). Five studies were published in peer‐reviewed journals, and the other two were PhD thesis in ProQuest. Out of seven studies, three were conducted in the United States of America, two in Denmark, one in Canada and one in Germany. The settings included surgery department (n = 2), outpatient oncology clinic (n = 1), Cancer Care website (n = 3) and cancer social group (n = 1).

**Table 1:** Brief overview of included studies

Author (Year)	Country	Setting	Sample size	Design	Disease	Data collection	Analysis methods	Brief findings
Engler et al (2016)	Germany	Oncological outpatient services of diverse hospitals in Berlin and Freiburg	60	Mixed design	Prostate, breast, colorectal cancer	Focus group discussion; one‐on‐one interview	Thematic analysis	Looking for similarity The importance of interviews being conducted in university settings under research leadership Format of website information and facilitating emotional response
Gates et al (2006)	USA	Cancer medical centre	6	Qualitative study	Breast, ovarian, testicular cancer	Unstructured interview	Narrative processes coding system; Riessman's approach to narrative inquiry	Remembering people Different perspectives, starring roles Getting more comfortable with cancer as a speed bump Don't suck with the same story Battle for my heart and mind Memories just stirred up emotions
Laing et al (2017)	Canada	AYA oncology community	16	Philosophical hermeneutic study	Paediatric/AYA cancer	Semi‐structured interview	Hermeneutic interpretation	Understanding: a profound need for others to know what it was like A brick in pathway to healing When it is least expected Truth and reconciliation
Gripsrud et al (2016)	USA	Breast and Endocrine Surgery in Stavanger University Hospital & Plastic Surgery Department of a National Cancer centre in the south‐western United States	7	Qualitative study	Breast cancer	Semi‐structured interview/	Thematic analysis	Writing as process telling my story Writing as a means to help others Writing as therapeutic A divergent case
Caldwell et al (2008)	USA	The Healing Journeys Cancer as a Turning Point (CTP) conference	12	Qualitative study	Cancer, chronic	Organic inquiry; semi‐structured interview	Thematic analysis	Transformative change in the participants Truth is a healing agent The feeling of being understood
Hoybye et al (2005)	Denmark	Breast cancer online resources	15	Ethnographic case‐study method	Breast cancer	Semi‐structured interview; participant observation	Content analysis	Empowerment through knowledge Tears and laughter Entering a new social world Social intimacy
Borregaard et al (2017)	Denmark	A Danish Cardiothoracic and Vascular surgery department	9	Qualitative study	Lung cancer	Narrative structured interview	Phenomenological hermeneutic analysis	Exchanging emotional thoughts is easier with a peer Talking to a peer reduces loneliness Being ambiguous about a relationship with fellow patients Being the main person in the conversation with a peer

### 3.3 Methodological quality of studies

**Figure 2:**
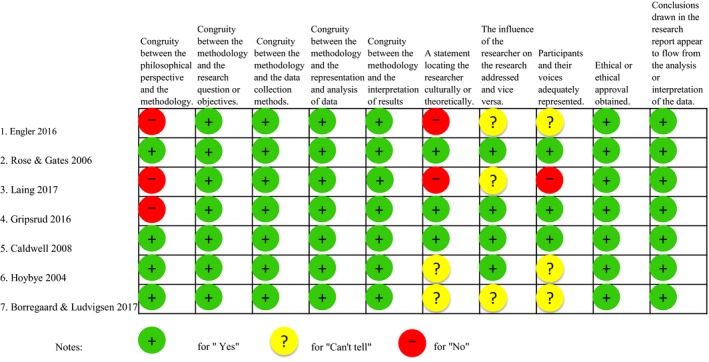
Critical appraisal by using JBI qualitative assessment tool

Both study design and content were considered to be of reasonable quality and of direct relevance to this review. Three studies did not present the philosophical perspective, two did not show the statement locating researcher culturally or theoretically and one did not absolutely present participants' voice (Figure 2).

### 3.4 Synthesis of main findings

Through meta‐synthesis of the findings of all the seven studies included in this systematic review, six themes were identified:

#### 3.4.1 Narrative practices reduce the gap between patients and clinicians in decision making

Reading, watching or listening to the story of a person similar to oneself was rated as especially helpful because upcoming decisions and challenges were seen to be highly variable within a disease…^24^

Patients thought narrative practice was a way to reduce the gap with clinicians in decision making. To patients, cancer treatment process is not a one‐and‐done journey. They will come across many issues to contemplate and decide during the course of treatment. Upcoming decisions and challenges vary within a disease. Reading, watching or listening to the story of a patient similar to oneself was particularly useful because patients could learn from the stories and their decision‐making process.^24^ At the end of narrative practice, patients could be aware that they really have some important issues to decide or deal with.^25^ The stories contain information and topics presented from different viewpoints with pros and cons. Patients like to read or see the reasons why people in the story refuse or accept a regimen. The content within the stories facilitates decision making.^24^ In patients' opinions, through telling stories to the health‐care professionals, they would gain a better understanding of what the patients went through.^25^, ^26^ As a result, the health providers will take into consideration patients' thoughts and feelings more often in the process of decision making.

While health providers know more about their patients, the patients eventually learn to trust their health providers more. This will narrow the gap between patients and clinicians. Thus, narrative practice plays an important role in patient‐clinician's relationship and decision‐making process.^23^, ^24^, ^25^

#### 3.4.2 Narrative practice is a way of healing

I can see the importance of my telling stories and hearing other people's stories because I think it's therapeutic on many levels.^25^

Narrative practice is a way to healing.^14^, ^16^, ^27^, ^28^, ^29^ Patients will gain hope, confidence, power, as well as motivation in the narrative practice.^10^, ^23^, ^24^, ^25^, ^27^, ^28^ Simultaneously, most of the patients feel released and comfortable after telling or writing their stories.^10^, ^25^, ^27^, ^28^ Therefore, telling, reading, listening or writing a story appears to have a significant therapeutic value. The process of narrative practice is undoubtedly therapeutic, but not a therapy.^26^

Patients could see the importance of telling stories and hearing other people's stories because of its therapeutic effect on many levels.^25^, ^28^ Storytelling or a chat is something that can heal, maybe not cancer but patients' souls.^14^ Writing their own story was therapeutic for patients. Without writing, they may get caught up in negative emotions, which may lead to mental illness.^10^ Many participants were convinced that they experienced a degree of healing related to their cancer experience as a result of writing their stories.^26^ The participants felt that they benefited dramatically from the stories at the story sharing conference and that they were unconstrained from fear, isolation, confusion, shame and self‐doubt.^28^

Most of the patients have a ‘wall’ in their mind to protect themselves from vulnerability. Nevertheless, narrative practice is a ‘brick to knock down the whole wall’. ‘When the wall comes down, the healing can happen’.^28^ Narrative practice is not the ‘cure’ to psychosocial recovery from cancer, but it can act like a bridge and can assist patients in moving forward in their lives.

#### 3.4.3 Narrative practice keeps patients in the social world

Shared laughter also establishes a shared social world, and on several occasions women on the mailing list said that they enjoyed talking to each other…Because we can laugh at the same things…The users described the breast cancer mailing list as a support group and a virtual community, a group notion that exists through shared communicative practices and social experience. 27

Narrative practice makes patients have the sense of presence in the social world. Each person is a member of the society. When faced with a cancer diagnosis, many patients feel like they have been abandoned. Listening to or telling stories about support and care was seen not only as reaching out to others in the society but also as a way of handling one's own experience and making it meaningful, leading to maintenance of self‐esteem.^27^ In patients' words, the story sharing conference made listeners feel a real connection with the storyteller, a friendship at a distance.^24^, ^28^ Sometimes, patients themselves are able to connect with each other without words after storytelling.^28^ Narrative practice makes patients feel that they are not alone, and they are loved.^10^, ^23^, ^27^ They could feel loving kindness that just holds them up, embraces them and keeps them warm.^23^ The multiple types of storytelling or story‐listening provided patients with a reconnection to a sense of community.^28^

Regarding the narrators, they believe narrative practice is a means to help and support others, and they desire to help others.^24^, ^26^ In the process of helping others, narrators, also cancer patients before, have a reconnection with other people and society.^28^ Narrative practice makes them more social and positive in the future life.^10^

#### 3.4.4 Narrative practice helps patients' reflection, self‐recognition and self‐realization

Participants reflected that this aspect of the expressive writing could be emotional and, at times, painful, but that it was also often affirming to reflect upon what they had been through, the choices they had made, and how much they had overcome.^10^

Narrative practice can make people reflect, recognize themselves and then realize the value of life.^10^, ^24^, ^25^, ^26^, ^28^

Firstly, narrative practice leads patients to reflect upon their own behaviour and thoughts.^24^ They could reflect on what they had gone through, the choices they had made, and how much they had overcome.^10^ When the patients read their own writings and stories, they would re‐assess the changes they have gone through.^28^ In other words, the stories helped them reflect upon the shift that they had made at that time and its continuous development in their life.^25^

Secondly, patients start to recognize themselves. Patients showed huge shifts as a result of listening to the stories.^28^ What's more, they represented big changes in mind about the nature of their own value and abilities.^10^ Many of the patients were able to accept what had happened to them and their experiences in this new light.^28^ Narrative practice opens up patients mind to what they are going through. It makes them recognize and understand what is happening to them.^10^, ^26^, ^28^

Finally, patients gained a new appreciation for themselves. Narrative practice made them generate the sense of helping others.^28^ This practice is about finding, facing or confronting a ‘truth’ from their experience with cancer. Once the truth was found, faced or confronted, the patients have already made sense of, or got the meaning from their experiences with cancer.^26^ They saw themselves in a new perspective. They are not only a person who simply survived, but also a person to utilize their knowledge to share with others, to help others go through the cancer journey and to achieve self‐realization.^10^, ^25^, ^27^, ^28^

#### 3.4.5 Narrative practice is likely to have negative effects on the patients

I'm good at kinda like ignoring I some of my worries and things I but when I get really down and then write things down I kinda find myself carried to the, like negative side and worry more.^16^

In addition to the positive effects, some reported that they also felt worried, nervous and overwhelmed at times.^14^, ^16^, ^27^, ^30^ Some people even got extremely depressed after telling their stories.^27^ They always reflected upon what they had been through, the choices they had made, and how much they had overcome. For others, it was painful because the process of storytelling or writing reminded them of their previous tough experiences.^16^ Because narrative practice brings out all those emotions and things that the patients do not really want to face, they felt uncomfortable and afraid at the beginning.^16^, ^27^

#### 3.4.6 Patients have different preferences on different approaches of narrative practice

Focus group discussions revealed that users searched for people in a similar situation and with similar characteristics to themselves. They looked for people of the same age, who had children of the same age, and/or for people who were comparable in terms of the time since diagnosis. Reading, watching or listening to the story of a person similar to oneself was rated as especially helpful because upcoming decisions and challenges were seen to be highly variable within a disease, so users tried to find those whom they could relate to the most.^24^

For narrators, they prefer telling stories in a private and safe place and writing stories at home.^10^, ^25^, ^28^ There are two different thoughts. Some people felt more comfortable talking.^25^ However, others did not feel comfortable when expressing to others.^10^ Although diverse thoughts may happen, both telling and writing stories still are two main approaches in narrative practice. The Internet served as a means to facilitate discussions. The Internet made it easier to start discussions on difficult and painful stories.^27^

In terms of sharing objects, people prefer talking to a peer, nurses or a social worker who helped them previously.^10^, ^23^, ^25^, ^28^ Many patients in the reviewed studies did not disclose to their families or friends as they did not want them to worry.^10^, ^23^ Sharing emotional thoughts is easier with a formal patient due to the similar experience and concerns.^23^ Nurses are professional care providers and know more about what the patient came through.^25^ Also, they have a background in caring for cancer patients, while an ordinary person may not have and so it is easier for the nurses to gain trust.^25^

Regarding the narrative content, patients are willing to read real and successful stories with humour.^23^, ^28^ Compared with the books, patients prefer video stories, which are more vivid and impressive.^24^ In addition, patients often look for similarities in narrative stories, for example, similar disease, similar age groups and similar regimens.^23^, ^24^, ^27^

## 4 DISCUSSION

The findings of this systematic review with meta‐synthesis regarding narrative practice in patient with cancer provide a deeper understanding of cancer patients' experiences. Seven studies were identified that met the inclusion criteria, and six themes regarding narrative practice were synthesized. The four main benefits of narrative practice patients with cancer experience include ‘it reduces the gap between patients and clinicians in decision making; it is a way of healing; it keeps patients connected with the social world; and it helps patients' reflection, self‐recognition and self‐realization’. Our findings also showed that narrative practice may have negative effects for some patients. The findings highlight that patients have different preferences regarding narrative approaches.

Our findings highlight the importance of narrative practice for patients with cancer. With significant increase in survival rates of patients with cancer over the last decades, more attention is required to understand how health‐care professionals can mitigate suffering and improve the quality of life of cancer patients.^31^ Some quantitative studies have identified the effectiveness of narrative practice in patients with chronic diseases.^14^, ^17^

Information sharing was found to be one of the six themes synthesized in this systematic review on the experience of patients with cancer regarding narrative practice. Studies have shown narrative practice is an effective approach through which people provide important messages and experiences to others.^30^ Many studies in different health‐care settings have shown that one of the main functions of narrative practice is to deliver information.^27^ The stories from narrative practice are not only helpful to patients with cancer to understand the process of illness and treatment decision, but also useful to health‐care professionals to provide care with empathy, reflection, professionalism and trustworthiness.^16^ Although other means to pass along messages exist, narratives continue to play an important role to bridge the gap between patients and health‐care professionals.^29^ This is because narratives are more engaging for patients, especially when they are relevant to them.^32^ In addition, information provided in narrative format is better retrieved and understood than information provided in other formats.^33^

This systematic review identified the therapeutic value of narrative practice. Narrative practices of storytelling, drawing and reading have been considered to have therapeutic use since the 1980s and have been used therapeutically in a variety of populations in diverse settings.^34^ Patients with cancer experience feelings of distress, humiliation, powerlessness and fear of dying.^35^ Studies have found that mental and physical health are mutually dependent, and the increase in psychosocial well‐being is associated with survival.^36^ In addition, as the life expectancy of patients with cancer has greatly increased due to the advances in cancer treatments, it is important to pay attention to the psychosocial effects of cancer and its treatments. Narrative practice is a potential psychosocial intervention to this vulnerable population. However, narrative practice is not intended to replace traditional psychological therapies and should be viewed as complementary to other therapies.

In this systematic review, we found that narrative practice helps to evoke the interactive relation and socialization aspects of patients with cancer. Patients in most of the studies which were included in this systematic review talked about their relationships with others, such as parents, spouses, children, friends, other patients with cancer or health‐care professionals. Patients with cancer sometimes feel they must conceal their worries in order to protect others, or they find it difficult to talk face to face with others about their feelings. Narrative practice allows patients to return to social connections with others and positively affect their relationships with others, by providing an opportunity to share their experiences.^10^ Thus, patients with cancer could progress from isolation to active participation in a new social context. Additionally, narrative practice empowers patients with cancer, as they perceive narrative as a way to help others. The active narrative practice, such as writing, storytelling and drawing, is movements for patients to transform their role from being acted upon to act upon.^10^, ^23^, ^27^ In narrative practice, patients feel they are performing for an imagined readership of patients with cancer. By narrating, they wish to provide peer support to the newly diagnosed cancer patients who can seek out and benefit from their narrative wisdom.

The synthesis in this review points out that narrative practice helps patients with self‐reflection, self‐recognition and self‐realization. During narrative practice, participants have the opportunity to organize their thoughts and experiences and express changes and effects, and it helps to distance themselves from situations.^37^ In addition, this practice helps patients to give meaning to their experiences of being diagnosed with cancer and their struggles.^37^ Narrative practice is a means to capture and articulate the many overwhelming and painful aspects of life after having cancer.^37^

In this systematic review, we also found the negative impacts of narrative practice on some patients with cancer. Some participants feel uncomfortable or even become depressed as narrative practice may bring out the emotions which they did not want to face. Therefore, health‐care professionals need to be aware and pay attention when utilizing narrative practice. Patients need not only treatments, but also caring. Health‐care professionals who view patients from a holistic perspective may see caring and healing as the goal of health care.

We also found ‘patients had different preferences on approaches of narrative practice’. This holistic approach is possible through acknowledging the benefits of narrative practice as a humanized way of psychological healing for cancer patients. To achieve this, firstly, the health‐care professionals need to be aware that narrative practice is similar to other psychological therapies and need to be trained to use this practice.^15^ Secondly, a tailored instruction is necessary to offer appropriately balanced suggestions and openings to allow the patients express their own thoughts, feelings and experiences.^10^ Lastly, health‐care providers should assess the preference and willingness of individual patients and select an appropriate narrative method. There is no ‘one method fits all’ approach of narrative practice. The implementation relies on patients' willingness, preferences, as well as surroundings.

Therefore, health‐care professionals need to be trained and prepared to conduct narrative practice to provide holistic care for the patient with cancer, taking into consideration their psychosocial needs, background, culture, preference, physical and psychological state. To the best our knowledge, this is the first systematic review to synthesize the experience of patients with cancer using narrative practice. This review followed the guidelines of systematic review and meta‐synthesis rigorously. We worked with the librarians to design a comprehensive search strategy and covered six English and three Chinese databases, as well as the reference list of identified articles. Finally, seven studies were included in this review (out of 2894), including four from North America and three from Europe. Thus, the possible limitation could be that some eligible studies in other countries and/or other languages may not have been identified.

## 5 CONCLUSION

Narrative practice is a humanized way for psychological healing in patients with cancer. Patients regard narrative practice as an approach for reducing the gap between patients and clinicians. This approach helps in decision making, is a way to healing, a method to keep patients in connected to social world, and provides a process for reflection, self‐recognition and self‐realization. However, individual patients with cancer have different and preferences regarding narrative practices. Additionally, it may have negative effects on some patients. Therefore, health‐care professionals need to be trained and prepared to conduct narrative practice. Consideration should be given to the background, culture, preference, physical and psychological state of patient with cancer.

## CONFLICT OF INTEREST

The authors declare they have no conflicts of interests.

## PROTOCOL REGISTRATION

This is a systematic review registered in PROSPERO (2017: CRD42017078900). URL: https://www.crd.york.ac.uk/prospero/display_record.php?RecordID=78900

## Data Availability

All data generated or analysed during this study are included in this published article and its Supporting Information files.
