# Supplementary material for: The experience of patients with cancer on narrative practice: A systematic review and meta‐synthesis
Source: Health Expect. 2020 Jan 16;23(2):274–83. doi: 10.1111/hex.13003 (PMC7104641; doi:10.1111/hex.13003)
Supplement: Supplementary file 1 [file HEX-23-274-s001.docx]

**Appendix**

Search Strategy

MEDLINE (Ovid): 1946 to August 23, 2017

Ovid MEDLINE(R) Epub Ahead of Print, In-Process & Other Non-Indexed Citations, Ovid MEDLINE(R) Daily and Ovid MEDLINE(R) 1946 to Present

| 1 | exp Narration/ | 6988 |
| --- | --- | --- |
| 2 | (narrative or storytelling).mp. | 22202 |
| 3 | exp Neoplasms/ | 3075717 |
| 4 | exp Oncology Nursing/ or exp Medical Oncology/ or exp Surgical Oncology/ or exp Radiation Oncology/ | 26348 |
| 5 | (cancer* or tumo* or malignan* or carcinoma* or oncolog*).mp. | 3157711 |
| 6 | (experience* or influence* or impact* or reflection* or perception*).mp. | 3102857 |
| 7 | 1 or 2 | 26355 |
| 8 | 3 or 4 | 4019939 |
| 9 | 5 and 6 and 7 | 988 |

Embase Classic+Embase 1947 to 2017 August 23

| 1 | exp storytelling/ | 832 |
| --- | --- | --- |
| 2 | exp narrative therapy/ or exp narrative/ | 6491 |
| 3 | (narrative or storytelling).mp. | 26291 |
| 4 | exp oncology/ or exp oncology nursing/ or exp oncology ward/ | 157316 |
| 5 | exp neoplasm/ | 3923273 |
| 6 | (cancer* or tumo* or malignan* or carcinoma* or oncolog*).mp. | 4541605 |
| 7 | (experience* or influence* or impact* or reflection* or perception*).mp. | 3817461 |
| 8 | 1 or 2 or 3 | 26291 |
| 9 | 4 or 5 or 6 | 5064260 |
| 10 | 7 and 8 and 9 | 1385 |

PsycINFO 1806 to 2017 August 23

| 1 | exp NARRATIVES/ | 17210 |
| --- | --- | --- |
| 2 | exp STORYTELLING/ or exp MUTUAL STORYTELLING TECHNIQUE/ | 4547 |
| 3 | (narrative or storytelling).mp. | 43701 |
| 4 | exp neoplasms/ | 44957 |
| 5 | exp ONCOLOGY/ | 3434 |
| 6 | (cancer* or tumo* or malignan* or carcinoma* or oncolog*).mp. | 71198 |
| 7 | (experience* or influence* or impact* or reflection* or perception*).mp. | 1434664 |
| 8 | 1 or 2 or 3 | 48322 |
| 9 | 4 or 5 or 6 | 73536 |
| 10 | 7 and 8 and 9 | 617 |
